# A retrospective data analysis on prevalence and risk factors for hypothermia among sick neonates at presentation to the neonatal intensive care unit of the Tamale Teaching Hospital

**DOI:** 10.1371/journal.pone.0303159

**Published:** 2024-05-16

**Authors:** Alhassan Abdul-Mumin, Naa Adzoa Adzeley Boi-Dsane, Samuel Tomilola Oladokun, Sheila Agyeiwaa Owusu, Patrick Ansah

**Affiliations:** 1 Department of Pediatrics and Child Health, School of Medicine, University for Development Studies, Box TL, 1350, Tamale, Ghana; 2 Department of Pediatrics and Child Health, Tamale Teaching Hospital, Box TL 16, Tamale, Ghana; 3 Navrongo Health Research Centre, Ghana Health Service, Navrongo, Ghana; University Medical Centre Ljubljana (UMCL) / Faculty of Medicine, University Ljubljana (FM,UL), SLOVENIA

## Abstract

Neonatal hypothermia, defined as an axillary temperature of <36.5C in a neonate, is common in neonatal intensive care units and is almost universal across all geographic and climatic regions of the world. This is even though environmental temperature is a known risk factor for its occurrence. We conducted a retrospective study in the Neonatal Intensive Care Unit of the Tamale Teaching Hospital (TTH) to document the prevalence and risk factors associated with hypothermia at presentation to the hospital. The study spanned the period from January 2019 to December 2019 and involved all neonates with axillary temperature documented at the time of admission. The prevalence of neonatal hypothermia in this study was 54.76%. Hypothermia was most common in neonates diagnosed with meconium aspiration syndrome (87/105, 82.86%), prematurity and low birth weight (575/702, 81.91%), and birth asphyxia (347/445, 77.98%). Neonates who were delivered vaginally were less likely to develop hypothermia compared to those delivered via Cesarean section. Inborn neonates (delivered in TTH) were 3.2 times more likely to be hypothermic when compared to those who were delivered at home. Neonates with low birth weight and APGAR scores < 7 at 1 and 5 minutes were more likely to be hypothermic. The dry season was found to be protective against hypothermia when compared to the rainy season. The overall mortality rate was 13.68% and the mortality in the subgroup with hypothermia at presentation was 18.87%. Our study documented a high prevalence of hypothermia with higher rates in neonates requiring intervention at birth. It is therefore crucial for perinatal care providers to adhere to the warm chain precautions around the time of birth.

## Introduction

Neonatal hypothermia is defined as the axillary temperature of a neonate <36.5°C [1]. Normal values for rectal temperature in both term and preterm neonates range from 36.5°C to 37.5°C [2]. This condition is common in neonatal intensive care units and is almost universal across all geographic and climatic regions of the world [3]. This is even though environmental temperature is a known risk factor for its occurrence [4,5].

A systematic review by Lunze et al. in 2013 on the neonatal hypothermia burden worldwide revealed that the condition is prevalent among both hospital births (32% to 85%) and births outside of the hospital (11% to 92%) irrespective of geographical location [6]. Two separate studies by Lunze et al. in 2013 and Mullany et al. in 2010 in Africa have reported prevalence rates >90%. Hypothermia might not often be a direct cause of neonatal mortality but contributes to mortality by compounding the patient’s condition. In one study in Nepal, it was documented that neonates born in the regions on the higher mountains with lower environmental temperatures were more likely to be hypothermic at presentation to healthcare facilities [5,6].

Most studies in sub-Saharan Africa on this topic are hospital-based and have reported a prevalence as high as 92% in some facilities [7–9]. One study from Guinea Bissau reported a very low prevalence compared to what has been reported in other studies in this region. The possible reason for this could be the definition used in the study (core temperature <34.5°C) [7], which was quite different from the known standard definition for hypothermia. A study on 136 neonates at admission to a neonatal unit in Ghana reported an admission prevalence of hypothermia (defined as axillary temperature <36°C) as 59% [10].

The determinants of hypothermia in neonates in Africa from previous studies include substandard thermal care practices, lack of education on thermal care of neonates, and lack of awareness at the community level. Low socioeconomic status, delivery outside a healthcare facility, bathing of neonates early, low birth weight, lack of knowledge among health workers, and delay in initiating breastfeeding were found to be the risks in the study conducted by Onalo in 2013 [7,11].

As a result of the risk factors, the World Health Organization (WHO) drew up guidelines to reduce neonatal hypothermia-related deaths termed ’the warm-chain guideline’. These measures include ensuring that the temperatures within delivery rooms are between 25°C to 28°C, drying the neonate immediately with towels, immediate initiation of skin-to-skin contact between the mother and neonate, keeping mother and neonate together, initiation of breastfeeding within an hour after birth, delayed weighing and bathing for at least 6 hours and 24 hours respectively, ensuring there is appropriate clothing and bedding *(with at least 3 layers of dry and absorbent material and a warm wrap)*, warm resuscitation *(using appropriate appliances during resuscitation)* and training of parents, caretakers and health workers in ways of reducing hypothermia [1].

Despite the universality of neonatal hypothermia and its contributions to morbidity and mortality, there is a dearth of information in Ghana on its prevalence. Hence this study aims to determine the prevalence and risk factors associated with hypothermia at admission to the Neonatal Intensive Care Unit (NICU) of the Tamale Teaching Hospital to assist in improving local practice among healthcare providers and planning for policymakers.

## Methods

### Study design and area

This is a hospital-based retrospective cross-sectional study conducted in the Neonatal Intensive Care Unit (NICU) of the TTH located in northern Ghana.

### Study area and period

This study was conducted in the NICU of TTH using data on admissions to the unit from 1^st^ January 2019 to 31^st^ December 2019. The hospital is situated in the Tamale Metropolitan Area, and it is a major referral site for four other regions in the Northern part of Ghana.

### The hospital

The hospital is the only tertiary hospital located in the northern part of Ghana, also serving as the teaching hospital for the University for Development Studies School of Medicine in Tamale. The NICU of the hospital is a 56-cot/incubator capacity unit with a Kangaroo Mother Care Unit attached to it. The unit receives and manages neonates with both surgical and medical conditions either born within the hospital or referred from other facilities within the catchment area. It is the only facility in the catchment area that provides advanced care for sick neonates. Thermal care in the unit is provided using 3 radiant warmers, 15 incubators, and KMC practiced in the KMC unit.

### Study population and sample size determination

The study population included all neonates admitted to the NICU of TTH during the study period regardless of the place of delivery. Neonates without a recorded axillary temperature on admission were excluded from the study. Using the single population proportion formula, a minimum of 322 patients were required for the study to be adequately powered. Our electronic database contained more than enough for this purpose (3169 admissions during the study period).

### Study variables

The dependent variable was neonatal hypothermia, and the independent variables were birth weight, age at admission, gestational age at delivery, the presence of birth asphyxia at delivery/need for resuscitation, APGAR score at 1 and 5 minutes after birth, place of delivery and mode of transport to hospital if referred.

**Operational definitions**

■ **Hot seasons:** February to May (for months of data collection only)
■ **Cold season:** June to September (for months of data collection only)
■ **Hypothermia on admission to NICU:** Axillary temperature of < 36.5°C measured during the initial assessment on admission to NICU.
■ **Birth weight is defined by WHO ((12)) as:**
  ○ Low birth weight < 2500g,
  ○ Very low birth weight <1500, and
  ○ Extremely low birth weight < 1000g

### Data collection procedure and tool

The NICU of the TTH keeps an electronic record of all admissions and outcomes of neonates admitted into the unit. This database was set up in 2018 to capture routine data at discharge. The study team designed a data collection sheet to include neonatal and maternal demographic information, source of admission, diagnosis for both morbidities and mortalities and duration of stay in the hospital. Specific neonatal information retrieved from the database includes birth weight, sex, mode of delivery, time of delivery, gestational age at delivery, APGAR score at one (1) and five (5) minutes, and place of delivery. The socio-demographic backgrounds of the mothers were also retrieved. Data collection began on the 13th of November 2020 through to the 8th of February 2021.

As part of the routine practice at the NICU, the axillary temperature and weight of the neonates were measured and recorded as the neonates arrived at the NICU. These parameters were entered into the electronic database. The ward head nurse, one supervisor, and the investigators supervised the data collectors closely during the entire data collection period.

### Data processing and analysis

All the entries were coded and entered into Epi Info version 7.1.2.0 and transferred to Stata 15 software for analysis. Descriptive statistics such as mean, percentage, and standard deviation were determined. Bi-variable logistic regression was performed to determine the association between each independent variable and the outcome variable. Variables with a P-value less than 0.25 in bi-variable logistic regression were entered into a multivariable logistic regression to adjust the effect of confounders on the outcome variable. The degree of association between dependent and independent variables was determined using the odds ratio with a 95% confidence interval. A P-value of less than 0.05 was considered significant.

### Ethics Statement

Ethical approval was granted by the Tamale Teaching Hospital Ethical Review Committee (TTHERC/30/09/20/05). Individual informed consent was waived for the conduct of this study due to its observational, retrospective, minimal-risk design. Additionally, there was no contact with patients or legal guardians as all data was obtained by study investigators through patient identification numbers on the electronic health system and subsequently fully anonymized to ensure patient confidentiality.

## Results

### Demographic information of participants

The majority 1340/2469 (54.63%) of the neonates included in the study were males. More than half 1550 (62.73%) of these were delivered by spontaneous vaginal delivery (SVD). 1449 (58.64%) of the neonates were inborn, 691 (27.96%) were outborn and 136 (5.50%) were delivered at home (Table 1).

**Table 1:** Demographic information of the neonates included in the study.

Variable	Frequency (%)
**Gender** Male Female	1350(54.63) 1119(45.29)
**Mode of delivery** SVD C/S	1550(62.73) 897(36.30)
**Place of delivery** Inborn Outborn Home Non-available	1449(58.64) 691(27.96) 136(5.50) 195(7.89)
**Day on admission** Day 1 Day 2 Day 3-Day 7 Above day 7	1521(61.55) 174(7.04) 347(14.04) 429(17.36)

### Baseline clinical information of the participants

Table 2 summarizes the baseline clinical information of the neonates included in the study.

**Table 2:** Baseline clinical information of participants.

Variable	Frequency (%)
**Birth Weight** Normal Low birth weight Very low birth weight Macrosomia	1240(58.38) 613(28.86) 213(10.03) 58(2.73)
**Temperature** Normal Hypothermia Fever	662(26.79) 1353(54.76) 456(18.45)
Mild hypothermia Moderate hypothermia Severe hypothermia	641(47.38) 709(52.40) 3(0.22)
**APGAR score at 1 minute** <7 ≥7	909(36.79) 1562(63.21)
**APGAR score at 5 minutes** <7 ≥7	382(15.46) 2089(84.54)
**Principal diagnosis** Birth Asphyxia Prematurity and LBW Neonatal infection Neonatal jaundice Congenital malformation Birth Injuries Seizures Hematological Disorders Meconium Aspiration Syndrome Macrosomia Others	445(18) 702(28.44) 567(22.97) 319(12.93) 162(6.56) 28(1.13) 27(1.09) 29(1.18) 105(4.25) 49(1.99) 35(1.42)
**Outcome** Discharged Died	2127(86.08) 338(13.68)

Over a quarter of the neonates were of low birth weight 613 (28.86%), and 213 (10.03%) were very low birth weight. More than half 1353 (54.76%) of the neonates were hypothermic at presentation to the NICU, and 456 (18.45%) presented with fever (axillary temperature ≥ 37.5°C). The rates of hypothermia were higher in inborn neonates (998/1449; 68.9%), compared to outborn babies (278/691; 40%).

909 (36.79%) of the neonates had an APGAR score at 1 minute of <7 and 382 (15.42%) had 5-minute APGAR score <7. The top five principal diagnoses and reasons for admission to the NICU were prematurity and LBW 7029 (28.44%), neonatal infection 567 (22.97%), birth asphyxia 445 (18.00%), neonatal jaundice 319 (12.93%), and congenital malformation 162 (6.56%). Most of the admitted neonates, 2127 (86.08%) were discharged successfully whilst 338 (13.68%) died during admission.

### Diagnosis and prevalence of hypothermia

As shown in Table 3, hypothermia was most prevalent among neonates with meconium aspiration syndrome 87/105 (82.86%) followed by prematurity and LBW 575/702(81.9) and birth asphyxia 347 (77.98%). However, in absolute numbers, there were more preterm and LBW neonates with hypothermia (575) than any other diagnosis.

**Table 3:** Prevalence of hypothermia according to diagnosis.

Diagnosis	Prevalence (%) of hypothermia
Birth Asphyxia	347/445(77.98)
Prematurity and LBW	575/702(81.91)
Neonatal infection	105/565(18.52)
Neonatal jaundice	91/319(28.53)
Congenital malformation	57/162(35.19)
Birth Injuries	17/28(60.71)
Seizures	8/27(29.63)
Hematological Disorders	11/29(37.93)
Meconium Aspiration Syndrome	87/105(82.86)
Macrosomia	32/49(65.31)
Others	20/35(57.14)

### Bivariate logistic regression

Beyond descriptive analysis, bivariate logistic regression was conducted to determine the strength of association between demographic and baseline characteristics, and neonatal hypothermia.

The results in Table 4 indicate that, except for sex, all the independent variables were found to be statistically associated with neonatal hypothermia. Neonates who were delivered by SVD were 40% less likely to develop hypothermia as compared to those who were delivered by C/S and the difference was statistically significant after adjusting for confounding variables in the model [OR = 0.59; (95%CI = 0.50–0.71); p<0.0001]. Similarly, neonates delivered in TTH (inborn) were 3.2 times more likely to be hypothermic as compared to those who were delivered at home and the difference was statistically significant after adjusting for confounding variables in the model [OR = 3.2; (95%CI = 2.26–4.60); p<0.0001]. In addition, neonates with birth weight ≥2.5 Kg were 70% less likely to have hypothermia as compared to those whose birth weight was <2.5 Kg, and the difference was statistically significant after adjusting for confounding variables in the model [OR = 0.31; (95%CI = 0.25–0.37); p<0.0001]. Furthermore, participants with APGAR scores≥7 and at 1 and 5 minutes had lesser odds of being hypothermic as compared to those with APGAR scores <7 at both 1 and 5 minutes respectively, and the difference was statistically significant after adjusting for confounding variables in the model [OR = 0.29; (95%CI = 0.24–0.35); p<0.0001], and [OR = 0.35; (95%CI = 0.27–0.45; p<0.0001] respectively.

**Table 4:** Bivariate logistic regression analysis of risk factors for neonatal hypothermia.

Variable	OR (95% CI)	p-value
**Gender** Female Male	Reference 0.85(0.72–1.01)	0.060
**Mode of delivery** C/S SVD	Reference 0.59 (0.50–0.71)	<0.000
**Place of delivery** Home Inborn Outborn	Reference 3.22 (2.26–4.60) 1.02 (.071–1.48)	<0.001 0.906
**Birth Weight (Kg)** <2.5 > = 2.5	Reference 0.31 (0.25–0.37)	<0.000
**APGAR score at 1 minute** <7 ≥7	Reference 0.29 (0.24–0.35)	<0.000
**APGAR score at 5 minutes** <7 ≥7	Reference 0.35 (0.27–0.45)	<0.000
**Season** Raining season Dry season	Reference 0.45 (0.38–0.53)	<0.001

### Multivariable logistic regression analysis

In the multiple logistic regression results, all the independent variables were found as predictors of hypothermia except sex, after adjusting for confounding variables in the model.

Neonates who were delivered by SVD were 28% less likely to have hypothermia at presentation to the NICU as compared to those who were delivered by C/S and the difference was statistically significant after adjusting for confounding variables in the model [AOR = 0.72; (95%CI = 0.58–089); p = 0.003]. Participants who were outborn were 64% less likely to develop hypothermia as compared to those who delivered at home and the difference was statistically significant after adjusting for confounding variables in the model [AOR = 0.36; (95%CI = 0.16–0.82); p = 0.014]. Similarly, neonates with birth weight ≥2.5 Kg were 72% less likely to have hypothermia as compared to those with low birth weight., and the difference was statistically significant after adjusting for confounding variables in the model [OR = 0.31; (95%CI = 0.25–0.37); p<0.001]. Finally, neonatal APGAR score of ≥7 and at 1 and 5 minutes was associated with a lesser likelihood of presenting with hypothermia as compared to those with lower APGAR scores [AOR = 0.37; (95%CI = 0.29–0.47); p<0.001], and [AOR = 0.56; (95%CI = 0.40–0.78; p = 0.001 respectively] (Table 5).

**Table 5:** Multivariable logistic regression of risk factors for neonatal hypothermia.

Variable	AOR (95%CI)	p-value
**Gender** Female Male	Reference 0.83(0.68–1.02)	0.081
**Mode of delivery** C/S SVD	Reference 0.72(0.58–0.89)	0.003
**Place of delivery** Home Inborn Out born	Reference 1.14(0.51–2.57) 0.36(0.16–0.82)	0.746 0.014
**Birth Weight (Kg)** <2.5 > = 2.5	Reference 0.28	<0.001
**APGAR score at 1 minute** <7 ≥7	Reference 0.37(0.29–0.47)	<0.001
**APGAR score at 5 minutes** <7 ≥7	Reference 0.56(0.40–0.78)	0.001
**Season** Raining season Dry season	Reference 0.41(0.34–0.51)	<0.001

The overall mortality in this cohort was 13.68% (Table 1). The mortality rate was higher among neonates who were hypothermic at presentation (18.87%) compared to those who were febrile (9.92%) or normothermic (6.52%). Using the chi-square test, there was a statistically significant association between mortality and temperature of participants (X^2^ = 71.29; p<0.001). The mortality was also higher for neonates presenting with moderate to severe hypothermia (27.60%) compared to those with mild hypothermia (9.14%) and this difference was statistically significant in the chi-square analysis (X^2^ = 66.00; p<0.001).

## Discussion

Neonatal hypothermia is common in healthcare settings across the globe and contributes significantly to morbidity and mortality. Our study found that more than 1 in every 2 neonates had hypothermia at presentation to the NICU. Although this rate is high, it is similar to findings from studies in different parts of the world [3,11,12]. Lunze et al. in a worldwide systematic review conducted in 2013 revealed the prevalence of neonatal hypothermia in hospitals to be between 32% and 85% regardless of geographical location [3]; a systematic review and meta-analysis done in Eastern Africa documented a neonatal hypothermia prevalence rate of 57.2% [12]; and a Ugandan study found a prevalence of 51% [12]. These studies point to the universality and high burden of hypothermia which require focused attention to reduce complications that may accompany it.

In our study, the most common diagnoses associated with hypothermia were meconium aspiration, preterm birth, birth asphyxia, and birth injuries as seen in Table 3. This finding is not different from previous studies, although it is generally thought that preterm babies are most at risk of developing hypothermia during the perinatal period [12]. The main reason behind the rates of hypothermia related to these diagnoses is the need for resuscitation in the delivery room or theatres after birth [7,13,14]. It is well documented that cold delivery rooms, surfaces, lack of warmers, and inability to start immediate skin-to-skin care in the delivery rooms break the warm chain and hence predispose neonates to hypothermia [1]. It is imperative, therefore, for perinatal care providers to adhere to the warm chain during resuscitation.

Several risk factors predispose a neonate to hypothermia; these include, but are not limited to, place of birth, preterm delivery, low birth weight, and low APGAR scores; however, the basic mechanism behind most of them is the non-adherence to the warm chain along the continuum of care [4,13,15,16]. Other risk factors of neonatal hypothermia that are equally important include delayed initiation of breastfeeding, non-practice of immediate skin-to-skin, need for resuscitation at birth, cold delivery room/operating rooms, placing babies on cold surfaces naked, delayed drying after birth, early bathing of babies, and other cultural practices and socio-economic conditions [7,13,14]. Like other studies [17,18], our study found in both the bivariable and multivariable logistic regression analysis that C/S delivery, being inborn, low birth weight, low APGAR scores at 1 and 5 minutes, and raining season births were significantly associated with neonatal hypothermia and these remained significant after adjusting for risk factors. The most plausible explanation for the relationship between low APGAR scores and hypothermia may be the failure of care providers to provide adequate warmth during resuscitation. This has been documented in a previous study by Tasew et al who reported that neonates with APGAR score <7 at 5 minutes were 3.7 times more likely to develop hypothermia [19,20].

Previous studies by, Lunze et al. and Mukunya et al showed that living in a tropical area is not protective against neonatal hypothermia [4,21]. Although our study corroborated these previous findings, we found a significant difference in rates between the dry and the rainy (wet) seasons in northern Ghana. Neonates born in the dry season were 41% less likely to develop hypothermia (p<0.01). During March and April, which forms the peak of the dry season in northern Ghana, environmental temperatures can be as high as 50 C in the afternoons and early evenings [22]. There is a need for further work regarding seasons, climatic conditions, and environmental factors concerning neonatal hypothermia in Africa.

The mortality rate among neonates with hypothermia in our study was 18.87% compared to the general mortality rate of 13.68% among all admitted neonates during the study period. Lunze et al. in their systematic review found global case fatality rates associated with neonatal hypothermia ranging from 8.5% to as high as 52% [3] while another study in Malawi recorded a higher rate of mortality associated with hypothermia [23]. Although it is difficult to document the direct contribution of hypothermia to deaths in these patients, it is well known that hypothermia can alter general metabolism in the neonate, predispose to hypoglycemia, increase oxygen consumption at the initial stages, depresses respiration, and have adverse effects on the functions of the central nervous system [7,24].

### Limitations

This study was conducted in a teaching hospital which serves as a major referral facility hence may not entirely represent community findings since more complicated cases are likely to be sent to tertiary hospitals for management. Also, this study was quantitative but a more comprehensive approach to determining the contributory factors to neonatal hypothermia taking into consideration the adherence to WHO guidelines would be to include a qualitative approach.

Furthermore, the study did not assess for other risk factors of neonatal hypothermia which include the time of delivery, time of initiating breastfeeding, neonatal pulse rate, initiation of KMC, maternal obstetric comorbidities, and risks before delivery. These factors were not assessed and therefore were not accounted for but could most likely be contributing factors to the prevalence of neonatal hypothermia, morbidity, and mortality.

The study did not document cord clamping time or compare rectal temperature (more representative of core body temperature) with the axillary temperature and was done in one hospital so there could not be any comparison of data with other teaching hospitals within the same country given that the time of the study and conditions may not be the same, but this would have been necessary for analysis in drawing relevant conclusions.

Finally, although the risk of death seemed to increase with increasing severity of hypothermia, it is difficult to infer if mortality was indeed from neonatal hypothermia alone or other associated diagnoses given that the other diagnoses could be contributory factors. Therefore, further studies and controls are warranted to make this distinction.

Despite these limitations, the study is the first of its kind in a tertiary facility in northern Ghana and hence provides significant data for both planning and practice.

The sample size was also large which increases the power of the study and the significance of findings which can be used to educate the public on risks, causes, and prevention of neonatal hypothermia to lower morbidity and mortality rates.

### Implications for practice and recommendations

The findings in this study show that there is a high prevalence of neonatal hypothermia among neonates admitted to the TTH NICU hence there is the need for healthcare workers to adhere strictly to the WHO warm chain guidelines including immediate initiation of KMC to help reduce morbidity and mortality. The fact that most of the neonates were delivered in healthcare facilities provides an opportunity for retraining and education of staff and caregivers on adherence to these guidelines to reduce rates of hypothermia in the hospital. As C/S delivery and the need for neonatal resuscitation increase the risks of neonatal hypothermia, perinatal care providers should ensure optimal thermal control during these procedures.

## Conclusion

There was a high prevalence (54.76%) of hypothermia among neonates in the TTH NICU. Meconium aspiration syndrome, prematurity, low birth weight, and birth asphyxia were the most common diagnoses associated with neonatal hypothermia. We found that vaginal delivery, home delivery, APGAR scores of 7 and above, and the dry season were all factors found to be protective factors against neonatal hypothermia. Mortality was significantly higher in neonates who presented with hypothermia. Based on these findings we recommend that perinatal care providers strictly adhere to the warm chain practices along the continuum of care to reduce the rates of hypothermia and its attendant complications. Refresher training for all cadre of staff along this continuum of care may improve practices.
